# More diversity in epithelial cell polarity: A fruit flies’ gut feeling

**DOI:** 10.1371/journal.pbio.3000082

**Published:** 2018-12-05

**Authors:** H-Arno J. Müller

**Affiliations:** Division of Developmental Genetics, Institute for Biology, University of Kassel, Kassel, Germany

## Abstract

Multicellular animals face the principle challenge to deal with two distinct compartments: the internal organismal compartment and the external environment. This challenge is met by the differentiation of cell sheets into epithelia, which provide a dynamic barrier in tissues, organs, and organisms. Cell polarity is key to all functions of epithelia, and compromising polarity causes many severe diseases. Within the past 20 years, research on *Drosophila melanogaster* discovered a conserved molecular machinery that controls epithelial polarity. Recent findings suggest that the textbook *Drosophila*-based paradigm of the control of epithelial polarity may not be as universal as previously assumed.

## Abstract 2

This Primer discusses recent research showing that the midgut epithelium of the adult fruit fly differs dramatically from the canonical modes of epithelial cell polarity in main features of its cytoarchitecture and its molecular control mechanisms.

## Introduction

Epithelial cells are the main building blocks of most organs in our body. Individual epithelial cells are characterized by a profound apical–basal polarity, which is central to their functions and the homoeostasis of tissues and organs. (The main structural features of polarized epithelial cells are described in Box 1.) Disruption of the apical–basal polarity results in dysfunctional epithelia and causes severe pathologies, like chronic inflammatory diseases, and is associated with the malignancy of tumors. The fruit fly, *Drosophila melanogaster*, has been one of the most influential model systems in the discovery of the molecular mechanisms that establish and maintain epithelial polarity. The key polarity regulators discovered in the fly turned out to be evolutionarily conserved and often serve similar functions in organisms as diverse as worms and mammals. Despite the similarities, the requirements of these conserved regulators for epithelial polarity are variable in the context of whole organisms. In the present issue of *PLOS Biology*, Chen and colleagues demonstrate that the conserved polarity machinery of *Drosophila* epithelia is dispensable in the *Drosophila* adult midgut epithelium and provide evidence for alternative pathways during polarization of epithelial cells [1].

**Box 1. Structural features of epithelial polarity in mammals and *Drosophila***

**Fig 1:**
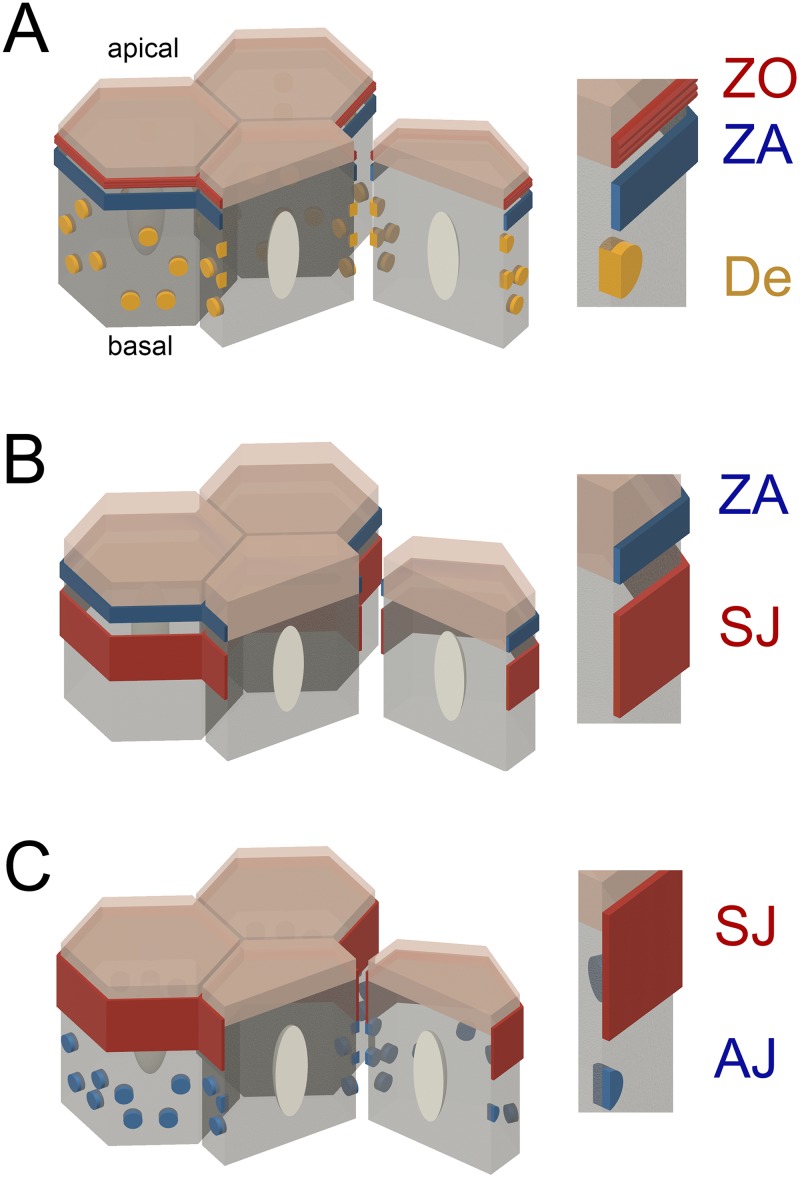
Composition and arrangement of intercellular junctions in mammalian and *Drosophila* epithelia. (A) Typical arrangement of the epithelial junctional complex in a monolayered mammalian epithelium. The apical membrane domain is labeled in transparent beige color and the basal–lateral membrane domain is labeled in gray color. The ZO (red) is localized apical to the ZA (blue). De (yellow) occur as spot-like junctions on the lateral interface of adjacent cells. (B) Arrangement of typical *Drosophila* epithelial junctional complex (apical domain, beige; basal–lateral domain, gray). The ZA (blue) forms the most apical junction followed basally by occluding SJs (red). This arrangement of junctional complexes is typical for ectodermally derived epithelia in *Drosophila*, except for the Malpighian tubules and the gastric caeca. (C) Arrangement of *Drosophila* epithelial junctional complex in the midgut epithelium. The SJ (red) is localized apical to AJs (blue), which are not organized into a belt-like ZA but occur as spot AJs throughout the basal area of the lateral membrane interfaces. AJ, adherens junction; De, desmosomes; SJ, septate junction; ZA, zonula adherens; ZO, zonula occludens.

Ultrastructural studies in the 1960s discovered a specific arrangement of intercellular junctions in mammalian epithelial cells, known as the epithelial junctional complex [2]. The junctional complex is essential for the structural integrity, the morphogenetic dynamics, and the communication between the apical and basal compartments of epithelial cells [3]. The epithelial junctional complex in mammals consists of the zonula occludens (ZO) (also known as tight junction), the zonula adherens (ZA) (also known as belt adherens junction), and desmosomes (also known as maculae adhaerentes) (Fig 1A). The ZO and the ZA form belt-like cell junctions that are typically juxtaposed to each other and are localized at the interface of the apical and the lateral plasma membrane domains. Desmosomes are spot-like contacts that are spread over the entire lateral plasma membrane domains of adjacent epithelial cells. The functions of intercellular junctions are diverse. The ZO controls the paracellular flux of compounds in between the cells through a branched network of sealing, strand-like membrane contacts. The key protein components of the ZO are tetraspan-transmembrane proteins of the claudin and the MAL and related proteins for vesicle trafficking and membrane link (MARVEL) families that associate with cytoplasmic plaques composed of ZO-1, Cingulin, and many other proteins [4]. The ZA is an adhesive structure in which the calcium-dependent cadherin/catenin system mediates a focus of intercellular adhesion [5]. The ZA is important for the dynamics and the remodeling of epithelia during the morphogenesis of developing and adult tissues [6]. Desmosomes are cell–cell attachment spots, to which the nonclassical cadherins desmoglein and desmocollin link intercellular adhesion with cytoskeletal intermediate filaments of the cytokeratin family through extensive cytoplasmic plaques containing plakoglobin, desmoplakin, and others [7].

The cytoarchitecture of invertebrate epithelial cell junctions is diverse among different species and exhibits distinct subtypes and compositions of intercellular junctions [8]. Within the present context, the focus will be on *Drosophila* epithelia. The typical intercellular junctional complex in most mature *Drosophila* epithelia includes an adherens junction of the zonula adherens (ZA) type and septate junctions (SJs) (Fig 1B). The SJs are occluding junctions that serve the restriction of paracellular transport across the epithelium and share similar molecular components with zonula occludens (ZO) from vertebrates, for example, the claudin family members Megatrachea, Sinuous, and Kunekune. In addition, important lateral regulators of epithelial cell polarity, including Scribble (Scrib), Discs large (Dlg), and Lethal giant larvae (Lgl), are localized to the SJ [9]. The structure of SJs, however, is different from ZOs, as areas of close membrane appositions are organized in ribbon-like septae between the lateral domains of adjacent cells. SJs come in many different flavors; *Drosophila* epithelia contain either pleated SJs (pSJs) or smooth SJs (sSJs). The difference between these two types of SJs is their ultrastructural appearance and their occurrence in distinct epithelial tissues. pSJs are typical for epithelia of ectodermal origin such as the hypodermis, the hindgut, and the trachea, while sSJs are present in epithelia of endodermal origin, like the midgut. The Malpighian tubules, despite being of ectodermal origin, also possess sSJs. Recently, three sSJ-specific proteins were identified: Snakeskin (Ssk), Mesh, and Tetraspanin 2A (Tsp2A) [10]. The ZA of *Drosophila* epithelia is very similar to the mammalian epithelial ZA in both its structural features, its molecular composition, and its functions [11]. Desmosomes or desmosomal proteins have not been described in *Drosophila*.

A major difference in mammalian epithelial compared to *Drosophila* epithelia is the localization of the intercellular junctions along the lateral cell interface of adjacent cells. While the ZO is apical to the ZA in mammals, in the fly, the ZA is apical to the SJ. Many epithelia in flies also exhibit a special cytocortical domain at the interface of the ZA and the apical membrane domain called the subapical region (SR) (also known as marginal zone). The SR does not have specific ultrastructural features but contains polarity protein complexes, like the Crumbs (Crb)/Stardust (Sdt) complex and the Partitioning defective (Par)/atypical protein kinase C (aPKC) complex, which are crucial in the controlling of epithelial polarity in embryogenesis [12].

## Intercellular junctions in the fly midgut

The presence of cell junctions at the interface of apical and basal plasma membrane domains is a universal feature of all epithelial cells (Box 1). The structure, the molecular compositions, and the relative arrangements of epithelial cell junctions along the apical–basal axis are different between vertebrates and invertebrates (Fig 1). In most *Drosophila* epithelia, the adherens junctions (ZA) are positioned apically to the occluding junction (SJ), while in mammals the occluding junction (tight junction or ZO) is positioned apically to the ZA (Fig 1A and 1B). Chen and colleagues demonstrate that the arrangement of cell junctions in the *Drosophila* adult midgut epithelium is distinct from other *Drosophila* epithelia; in the midgut, the SJs form apical to the adherens junctions (Fig 1C) [1]. Thus, the cytoarchitecture of the *Drosophila* midgut epithelium appears more similar to vertebrate epithelia than to other epithelia in the fly. These data confirm and extend ultrastructural studies, which demonstrated that in epithelia derived from the endoderm germ layer, sSJs (Box 1) are apical to more diffusely distributed spot adherens junctions [9, 13] (Fig 1C). The presence of sSJ is unlikely to be the cause of the distinct apical–basal arrangement of cell junctions in the midgut because other epithelia harboring sSJs, like the Malpighian tubules, exhibit the canonical arrangement of intercellular junctions.

## Conserved polarity regulators in the fly midgut: Dismissed

Genetic screens in the nematode *Caenorhabditis elegans* and in *Drosophila* have identified the key players in the control of epithelial polarity. These studies revealed that epithelial cell polarity in the fly is controlled by three plasma membrane–associated protein complexes, often called “canonical polarity complexes”: two apical protein complexes, the Crb/Sdt complex and the Par/aPKC complex, and one lateral protein complex [12, 14]. The Crb/Sdt complex is composed of the transmembrane protein Crb and its cytoplasmic binding partners including the scaffolding protein Sdt. The central components of the Par/aPKC complex are the scaffolding proteins Bazooka (Baz)/Par3 and Par6 and aPKC. The lateral protein complex consists of the scaffolding proteins Scrib, Dlg, and Lgl. Elegant genetic studies demonstrated that the positioning of the ZA depends on a mutual antagonism between the apical and lateral protein complexes [15, 16]. Importantly, all of the core polarity proteins discovered in *Drosophila* and *C*. *elegans* are highly conserved, but despite their evolutionary conservation, mutations in polarity genes in mammalian organisms rarely exhibited epithelial polarity phenotypes that were similarly striking as in *Drosophila*. The present study by Chen now discovered that the polarity of the adult fly midgut epithelium does not require the canonical polarity regulators. The polarity of the midgut epithelium was not affected by depletion of the apical Par6 and aPKC proteins or depletion of the lateral proteins Scrib, Lgl, or Dlg. Expression of the important polarity genes *crb* and *baz* was not even detectable in the midgut. This surprising result raised the question of how polarity is generated in the adult fly midgut.

## Polarity control in the adult *Drosophila* midgut: From the bottom to the top

In the absence of a polarity control mechanism by the canonical apical and lateral polarity genes, Chen and colleagues considered the basal membrane domain as a host of a possible polarity cue. The basal domain of differentiated epithelia interacts with the extracellular matrix (ECM) via receptors of the integrin family [17]. Chen and colleagues found that depletion of Talin, encoded by the *Drosophila rhea* gene and a central component of the integrin adhesive machinery, causes the failure of midgut cells to polarize and to establish proper SJs. This result suggested that the formation of SJ was downstream of the basal cue through integrins, and the authors therefore tested whether sSJ resident proteins were important for formation of the apical domain. Mutational analyses of the sSJ components Mesh and Tsp2a showed that the ability of the new-born enterocytes to integrate into the epithelium was dependent on the formation of SJ. In conclusion, the mechanism of enterocyte polarization in the adult midgut depends on basal cues from integrin-mediated adhesion acting upstream of the formation of SJ and the generation of an apical membrane domain. This finding is important, as it contrasts with the previously known mechanisms of epithelial polarity in the fly and provides a solid base to discover alternative mechanisms using the power of *Drosophila* genetics.

## An alternative route for epithelial polarization

Why is the adult midgut epithelium so different in its structure and molecular control to other epithelia in the fly? One reason may be that the midgut epithelium is derived from the endoderm while most other epithelia in the fly are derived from the ectoderm that ultimately originate from the blastoderm epithelium. A germ layer–specific regulation of epithelial polarity has recently been discovered in the diploblastic cnidarian *Nematostella vectensis* [18]. This opens the interesting possibility that there might be distinct and evolutionarily conserved genetic programs for the establishment of epithelia derived from ectodermal versus endodermal origin. The midgut epithelium originates from two ectoderm precursors in the embryo: the anterior and the posterior midgut primordia. These primordia undergo an epithelial mesenchymal transition (EMT) [19], migrate toward each other, and then undergo a mesenchymal epithelial transition (MET) in which they reform an epithelium and, together with the anterior and posterior midgut epithelia, generate a continuous intestinal tube in the late embryo. The midgut cells differ from the foregut and hindgut, as they never express the polarity gene *crb* and do not form apical adherens junctions [13, 20]. The GATA family transcription factor Serpent is essential for the EMT of the midgut primordia, and integrin adhesions are required for the migration of the midgut precursor cells after EMT [21–24]. However, the genetic basis of the MET and the factors that are required for the polarization of the midgut in the embryo remain largely obscure.

What is the origin of the adult midgut? The adult midgut is derived from progenitor cells, called adult midgut progenitors (AMPs), that are set aside from the midgut primordia during late embryonic development [19, 25]. These progenitors form nests in which they proliferate in late larval and pupal development and generate the adult midgut epithelium during pupariation [26]. Some of the AMPs remain associated with the basal–lateral domain of the adult midgut epithelium as intestinal stem cells (ISCs), which are capable of dividing and differentiating into enterocytes to support the homeostasis of the tissue [27]. The genetic control of the initial adult midgut morphogenesis during metamorphosis is not well investigated, and it will be interesting to see how far the data reported by Chen and colleagues will apply to the polarization of AMPs in the pupa.

Chen and colleagues target their mutational analysis to the integration of ISCs into the established midgut epithelium. Therefore, one possible explanation of the principle difference in the epithelial polarity of the midgut could be that the mechanism applies exclusively to the ISC-to-enterocyte differentiation. This raises the question whether this way of polarization during differentiation of epithelial stem cell progenies is conserved. It has been known for a long time that basal cues are important for epithelial polarization and the orientation of the apical–basal axis in mammalian epithelial cells [28]. In cultured epithelial cells, the position of the ECM controls the polarity axis and the morphogenesis of epithelial cysts [29, 30]. During mammalian kidney development, the deposition of laminin A plays a crucial role in the MET of the metanephric mesenchyme [31]. Thus, it may be that in a context in which epithelial tissues are derived from mesenchymal cells or stem cells, basal cues are overwhelming and apical cues become downstream effectors of such basal cues [32]. The ISCs in the midgut epithelium might have maintained characteristics from such MET behavior of the larval AMPs in the sense that the ISCs are rudiments of embryonic mesenchymal cell reservoirs that can be activated when there is need for turnover. It will therefore be very interesting to see how the basal cues are interpreted through cell signaling and cell mechanics to translate into the establishment of the apical domain. Since aggressive tumors are capable of undergoing several rounds of EMT/MET, the mechanistic understanding of MET will be important in providing opportunities for the treatment of cancer progression.

## Abbreviations

AMP: adult midgut progenitor
aPKC: atypical protein kinase C
Baz: Bazooka
Crb: Crumbs
Dlg: Discs large
EMT: epithelial mesenchymal transition
ISC: intestinal stem cell
Lgl: Lethal giant larvae
MARVEL: MAL and related proteins for vesicle trafficking and membrane link
MET: mesenchymal epithelial transition
Par: partitioning defective
pSJ: pleated SJ
Scrib: Scribble
Sdt: Stardust
SJ: septate junction
sSJ: smooth SJ
Ssk: Snakeskin
Tsp2A: Tetraspanin 2A
ZA: zonula adherens
ZO: zonula occludens
